# What can traditional plant therapy do in the face of Covid-19? Examples from traditional Chinese medicine

**DOI:** 10.4314/ahs.v23i2.7

**Published:** 2023-06

**Authors:** Dandan Song, Qian Deng, Hualiang Chen

**Affiliations:** Department of Pharmacy, Shaoxing Seventh People's Hospital, Shaoxing, Zhejiang, China

**Keywords:** COVID-19, traditional plant therapy, Qingfei Paidu decoction

## Abstract

**Background:**

Until June 2022, more than 540.9 million people had been diagnosed with COVID-19, and the pandemic had claimed more than six million lives worldwide. Two years after fighting the virus, we faced a more uncertain position. SARS-CoV-2 is constantly mutating and reappears regularly, particularly with Omicron variants showing high genetic variation and immune escape mechanisms. The efficacy and duration of protection of existing vaccines against new variants of SARS-CoV-2 remains uncertain. The world needs time to develop new variant-specific drugs, including monoclonal topics, vaccines, and other antiviral drugs, to fight the epidemic.

**Objective:**

The aim of this study was to illustrate the scientific, effective and systematic nature of three classical prescriptions of traditional Chinese medicine (TCM) for the treatment of COVID-19 through comparison of disease symptoms, diagnostic process, and treatment methods and evidence-based and pharmacological studies.

**Methods:**

We analysed the “Diagnosis and Treatment Protocol for Novel Coronavirus Pneumonia” (Version 9) made by China, “WHO-2019-nCoV-therapeutics”, “Therapeutic Guidelines” published by Australian Therapeutic Guidelines Limited, “Shanghan Lun (Treatise on Febrile Diseases)”, “Jinkui Yaolue (Golden Chamber Synopsis), and “Wenyi Lun (The Epidemic Febrile Disease)”. We manually retrieved the dictionary of traditional Chinese medicine (Version II). In addition, we searched the Wiley online library, National Center for Biotechnology Information (NCBI), VIP, WHO website, and China National Knowledge Infrastructure (CNKI) for relevant literature from 2001 to 2022. We searched the original plants, ingredients, pharmacology, functions and indications, usage and dosage, drug efficacy, literature sources, and conduct an evidence-based studies. We quantified the strength of pharmacological action to show the pertinence of disease development.

**Results:**

We found that the diagnosis and treatment of pulmonary infection caused by epidemic disease in TCM classics is consistent with the diagnostic process of modern medical therapeutic guidelines. The three classic prescriptions have significant symptomatic therapeutic effects on the respiratory, gastrointestinal, urinary and hematological symptoms of the clinical manifestations of COVID-19. It was found that the herbal functional group of Houpo *(Cortex Magnoliae Officinalis*), Chaihu (*Radix Bupleuri*), Cangzhu (*Rhizoma Atractylodis*), Qianghuo (*Notopterygii Rhizoma et Radix*), etc showed strong anti-inflammatory activity and had a positive effect on treating and preventing the outbreaks of systemic inflammatory factors.

**Conclusion:**

TCM can obtain obvious curative effect in symptomatic treatment, has strong anti-inflammatory effect, and can effectively reduce symptoms and patients' pain.

## Introduction

SARS-CoV-2 is constantly mutating and reappears regularly, particularly with Omicron variants showing high genetic variation and immune escape mechanisms, which may negate the protection of the vaccine and lead to reinfection^1^. At the same time, the high transmission rate of Omicron is accompanied by an exponential increase in infection between people as well as in the mortality rate of infected individuals, rapidly overwhelming existing healthcare capacity. Infections are growing exponentially and rapidly outpacing existing medical capabilities^2^. All established methods of isolation, vaccination, discovered therapeutic antibodies^3^ and treatments seem helpless in the face of the Omicron variant. The world needs time to develop new variant-specific monoclonal antibodies, new drugs, and new vaccines to fight against the epidemic.

This study aims to explain the scientific strength, and effectiveness of the three classic prescriptions of TCM for the treatment of COVID-19, by comparing symptoms, diagnostic process, treatments, quantifying the intensity of pharmacological action and evidence-based pharmacology research. We hope that TCM may play an important role in filling in the gap of vaccine and drug treatment of the SARS-COV-2 Omicron (BA.1 and BA.2).

## Materials and Methods

### Materials

We analysed the documents about COVID-19 treatment, including the “Diagnosis and Treatment Protocol for Novel Coronavirus Pneumonia” (Version 9) made by China and “WHO-2019-nCoV-therapeutics”; “Therapeutic Guidelines: Respiratory (Version 5)”; “Therapeutic Guidelines: Gastrointestinal (Version 5)”: Functional gastrointestinal disease; “Therapeutic Guidelines: Rheumatology (Version 3)”; “Therapeutic Guidelines: Cardiovascular (Version 6)” ; “Therapeutic Guidelines: Endocrinology (Version 5)” and “Therapeutic Guidelines: Antibiotic (Version 15)”: Gastrointestinal infection: acute gastroenteritis published by Australian Therapeutic Guidelines Limited.

Current research analysed the classical literature of “Qingfei Paidu Decoction”, “Huashi Baidu Decoction” and “Hanshi Zufei Decoction” including “Shanghan Lun (Treatise on Febrile Diseases)”, “Jinkui Yaolue (Golden Chamber Synopsis), and “Wenyi Lun (The Epidemic Febrile Disease)”. Besides, the “Criteria of diagnosis and therapeutic of diseases and syndromes in traditional Chinese medicine” made by the National Administration of Traditional Chinese Medicine was analysed.

The basic decoction pieces were retrieved in the dictionary of traditional Chinese medicine (Version II) manually. The version of the classical literature and the dictionary was described in detail in our previous studies 4-6. We also searched the relevant literature from 2001 to 2022 in Wiley online library, National Center for Biotechnology Information (NCBI), VIP, the WHO website and China National Knowledge Infrastructure (CNKI). We searched the original plants, ingredients, pharmacology, functions and indications, usage and dosage, drug efficacy, literature sources, and conduct an evidence-based studies.

### Methods

This study analysed the respiratory, gastrointestinal, cardiovascular, and hematologic symptoms caused by COVID-19, and the comparative studies refer to pathogenesis, symptomatic diagnosis, clinical manifestations, pathological grouping (changes in the main cases), laboratory tests, pharmacology, (changes in the main cases) and therapeutic methods of TCM and western medicine. By comparing different academic ideas, the theory of formula and drug composition was investigated. We summarize and document the search results.

According to the three classical prescriptions, we performed pharmacological queries for each Chinese herb, conducted the original literature verification for each data of pharmacological action, and verified the original literature of pharmacological experiments. We validated the effective chemical composition of each Chinese herbal decoction. The original literature of clinical trials and clinical treatments in the form of prescription was searched. Each pharmacological action, important chemical composition and content were recorded, and the strength of the pharmacological action was marked and checked.

In order to facilitate digital statistics and analysis, we measured the strength of pharmacology by grade intensity. We recorded the intensity (frequency) of the main pharmacological action of the conventional dose of TCM as 1, and recorded intensity of very strong pharmacological effect as 5 in pharmacological experiments. The pharmacological effect of TCM which was strong but not very strong was recorded as 2 or 3. The intensity of high content of active ingredient with strong pharmacological effect was recorded as 4. The system frequency is the sum of pharmacological effects of the Chinese herbals on a certain part or system of the body.

The data was collected and analysed using Excel software (Microsoft, USA).

## Results

### Comparative studies on the diagnosis process and classification of disease of TCM and western medicine

Comparative studies have found that the diagnosis and treatment process of lung infection in TCM classics is actually consistent with the therapeutic guidelines of modern medicine. There is a certain corresponding relationship between TCM syndrome types and western medicine disease types.

Syndrome classification in TCM usually reflects a time period of disease development. TCM summarizes the current clinical symptoms and physical symptoms by observing, smelling, inquiring, and palpating, reflecting the summary (syndrome) of clinical symptoms and physical symptoms with the characteristics of TCM, which is actually the classification of diseases.

The general classification of diseases in western medicine mainly reflects the results of disease development, but also reflects the causes and symptoms of diseases. The clinical evaluation and diagnosis of western medicine is also a dialectical process of the disease. The “'treatment guidelines” for disease and disease classification are also temporal cross-sections of disease progression. As shown in Table 1, it can be seen that both Chinese and Western medicine are opened by the treatment measures taken after diagnosis and judgment of the etiology and condition of the disease. However, the pharmacological effects of the formulations were generally consistent and the efficacy was expected to be consistent (Table 1).

**Table 1:** Comparison of TCM and Western Medicine on respiratory symptoms of COVID-19

WHO-2019-nCoV-therapeutics			Diagnosis and Treatment Protocol for Novel Coronavirus Pneumonia (Version 9)	Chinese Medical treatment guidelines for COVID-19	
Clinical classification and clinical symptoms			Clinical classification and clinical symptoms associated with the Chinese protocol	Symptoms described in TCM classics Shanghan Lun (Treatise on Febrile Diseases) and Jinkui Yaolue (Synopsis of Golden Chamber)	Treatment options
Mild case		Symptomatic patients conform to the COVID-19 case definition without evidence of viral pneumonia or hypoxia	Mild case: The clinical symptoms were mild, and no pneumonia was observed on imaging.	The early performance: Taiyang Disease-Pulse floating, head and neck strong pain, aversion to cold.	Mahuang Xingren Shigao Gancao Decoction

Ordinary case	Pneumonia	Adolescents or adults with clinical signs of pneumonia (fever, cough, dyspnea, shortness of breath), but no signs of severe pneumonia, including SpO2 ≥ 90% under indoor conditions. Children present with clinical signs of non-severe pneumonia (cough or dyspnea + tachypnea and/or chest depression), but no signs of severe pneumonia	Ordinary case: With the above clinical manifestations, imaging findings of pneumonia.	Mild: 1. Taiyang disease: sweating after the great sweat, the stomach is dry and restless, if want drink, drink little water to make stomach comfortable. Pulse floating, urination unfavourable, mild heat, thirst.2, Taiyang disease: sweating already, pulse floating still, thirsty.	Wuling Powder
Severe case	Severe pneumonia	Clinical signs of pneumonia (fever, cough, dyspnea, tachypnea) in an adolescent or adult + one of the following Respiration rate >30 times/min; severe dyspnea; or SpO2<90% under indoor conditions. Children with clinical signs of pneumonia (cough or dyspnea) + at least one of the following: Central cyanosis or SpO2 <90%; severe dyspnea (e.g., shortness of breath, snoring, very severe chest depression). Common danger symptoms: refusal to milk or drink, drowsiness or coma, or convulsions.	Severe case: Adults conform to any of the following criteria: 1. Shortness of breath, RR≥30 times/min; 2. At rest, oxygen saturation ≤93% when inhaling air; 3. Arterial partial pressure of oxygen (PaO2)/oxygen concentration (FiO2) ≤300mmHg (1mmHg=0.133kPa); PaO2/FiO2 should be calibrated according to the following formula for high altitudes (above 1000m): PaO2/FiO2× [760/atmospheric pressure (mmHg)]. 4.The clinical symptoms were progressively aggravated, and lung imaging showed significant lesion progression >50% within 24 ~ 48 hours. The children conform to any of the following criteria:1.High fever lasting more than 3 days; 2.Shortness of breath (< 2 months, RR≥60 times/min; From 2 to 12 months, RR ≥50 times/min; From 1 to 5 years old, RR≥40 times/min; > 5 years old, RR≥30 times/min), Except the influence of fever and crying; 3.At rest, oxygen saturation ≤93% when inhaling air ; 4.Assisted breathing (nasal flapping and three depressions sign); 5. Lethargy and convulsion; 6. Refuse to feed or feeding difficulty, dehydration sign	Mild-moderate. Typhoid fever five or six days, Taiyang disease causes fever and sweat, do not wish to diet upset to vomiting or not vomiting, abdominal pain, unfavourable urination, mild fever and cough.	Xiaochaihu Decoction
Critical illness case	Acute respiratory distress	Onset: new respiratory symptoms or exacerbation of existing respiratory symptoms within one	Critical illness case: In one of the following circumstances:1. Respiratory failure requiring mechanical ventilation; 2. Shock	moderate-severe: Pulmonarycarbuncle, cough and reversed flow of qi, frog sound in throat.	Shegan Mahuang Decoction
Critical illness case	Acute respiratory distress syndrome (ARDS)	Chest imaging: (X-ray, CT scan, or lung ultrasound): Opacity of both lungs that cannot be fully explained by lung volume overload, lung lobe or lung atrophy, or pulmonary nodules. Causes of lung infiltration: Respiratory failure that cannot be fully explained by heart failure or excess fluid.	Critical illness case: In one of the following circumstances:1. Respiratory failure requiring mechanical ventilation; 2. Shock 3.ICU care is required for other organ failure	moderate-severe: Pulmonarycarbuncle, gasping cannot lay down.	Tingli Daozao Xiefei Decoction
		If there are no risk factors, an objective evaluation (e.g., echocardiography) should be performed to rule out hydrostatic pressure induced lung infiltration/edema. Oxygenation disorder in adults			

### Evidence-based results of pharmacological effects of TCM prescriptions

Evidence-based studies have found clear experimental pharmacological evidence to support the prescriptions for COVID-19 treatment in Chinese medical classics. The components of TCM showed clear and effective pharmacological activity in the form of a therapeutic group and produced corresponding therapeutic effects. In the form of pharmacological groups, the three traditional Chinese herbal formulas of this study were in the following categories: thermogenesis^7^, sweating^8^, ^9^, relieving cough^10^, ^11^, expectorant^12^-^14^, relieving asthma^9^, ^15^, analgesia^16^, antipyretic effect^15^, anti-inflammatory^13^, ^16^-^21^, anti-tumor^22^-^25^, antibacterial^26^-^29^, antiviral^27^, ^30^, ^31^, promoting digestive juices secretion, promoting gastric emptying^32^, promoting gastrointestinal movement^33^-^35^, repairing stomach injury, repairing intestinal injury^36^, ^37^, liver protection^38^-^42^, anti-diarrhea^37^, antiemetic effect^43^, cardiotonic effect^44^, myocardial protection, anti-platelet aggregation^45^, promoting blood circulation, anti-arrhythmic effect^46^, anti-cholinergic effect, anti-allergy^47^, antidepressant like effect^48^, diuresis, reducing blood sugar^49^, etc. There is clear pharmacological activity and evidence to support clear pharmacodynamics.

Pharmacological effects were quantified on a scale of 1-5. The statistical data analysed by graphs showed that the main pharmacological effects of Chinese herbal formulas were centrally distributed. This distribution trend directly shows that the components of TCM function in the form of pharmacological groups. The form of the pharmacological group reflects the therapeutic value of TCM, which intuitively shows the main and concurrent effects of TCM prescriptions in treating diseases. After quantitative analysis, the therapeutic effect of TCM can be clearly presented. Quantitative results are presented graphically. (Figure 1)

**Figure 1 (a):**
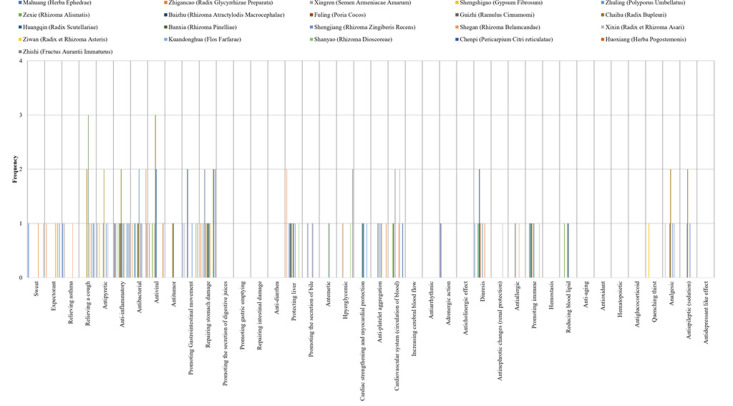
The pharmacological basis of Qingfei Paidu Decoction

**Figure 1 (b):**
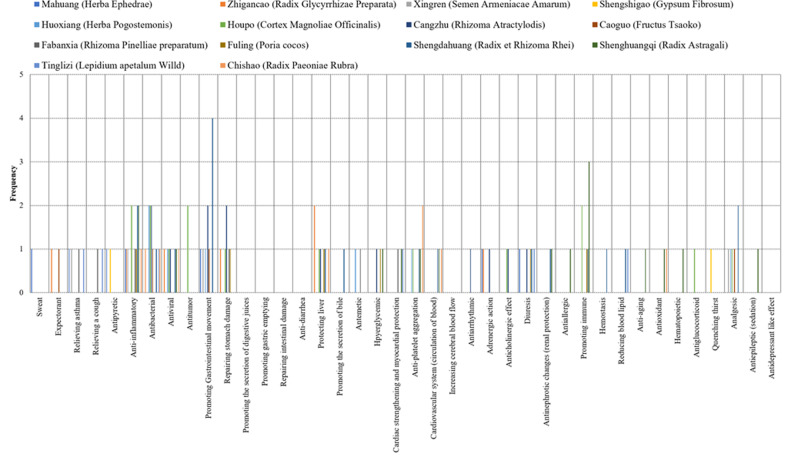
The pharmacological basis of Huashi Baidu Decoction

**Figure 1 (c):**
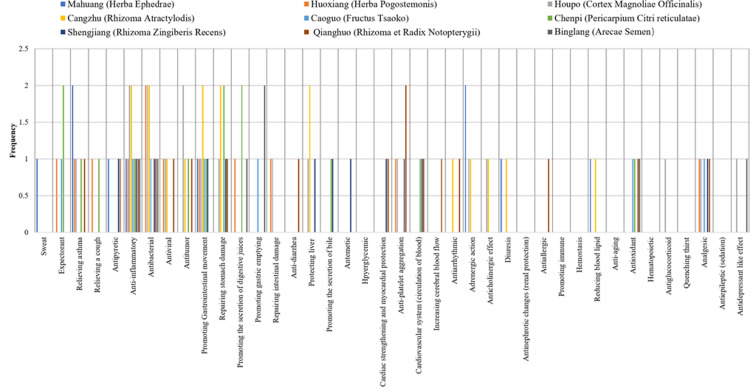
The pharmacological basis of Hanshi Zufei Decoction

### Evidence-based results of the relationship between laboratory biochemistry indicators of important diseases and the pharmacological action of TCM prescriptions

**Figure 2:**
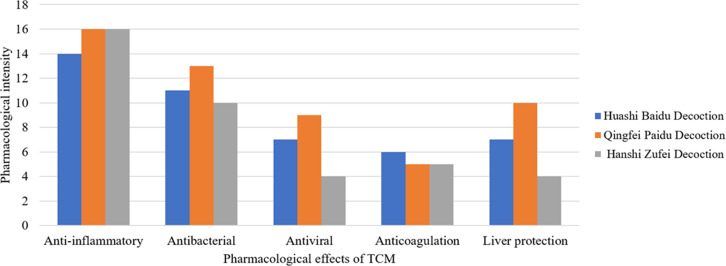
Comparison between important laboratory parameters of COVID-19 and functional groups of TCM with clear pharmacological support

The three classical TCM prescriptions in the form of pharmacological groups have clear basic experimental support for the important laboratory parameters of COVID-19, and the results showed that they have clear and powerful functional activities, including anti-inflammatoryt^50^, ^51^, antibacterial, liver protectiont^52^, ^53^, clear antiviral and anticoagulant effects, and also have clear pharmacological activity and molecular pharmacological evidence support. (Figure 2) and (Table S1).

**Table S1:** Comparison between important laboratory parameters of COVID-19 and functional groups of TCM with clear pharmacological support

Laboratory parameters	Pathology	Pharmacological effects of TCM	Pharmacological intensity of the components of TCM in Qingfei Paidu Decoction	Pharmacological intensity of the components of TCM in Huashi Baidu Decoction	Pharmacological intensity of the components of TCM in Hanshi Zufei Decoction
CRP↑					
RCT↑	Persistent	Anti-inflammatory	14	12	11
WBC↑	inflammation				
TNF↑		Antineoplastic	2	2	5
Calcitonin↑	Bacterial infection	Antibacterial	13	11	10
D-Dimer↑	Thrombotic disease	Anticoagulation	5	6	5
Liver enzyme↑ LDH↑	Hepatic injury	Liver protection	10	7	4
	Virus infection	Antiviral	9	7	4

Note: The Pharmacological intensity is the sum of the respective components of the three TCM prescriptions.

### Evidence-based results of the relationship between the pharmacological effects of TCM on important tissues and the corresponding symptoms

**Figure 3:**
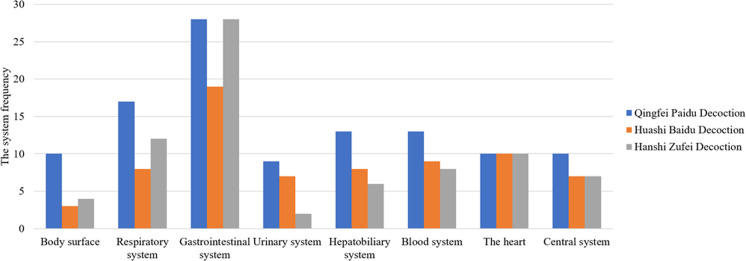
Chinese herbal slices with corresponding pharmacological effects for symptoms and vital organs of COVID-19

Confirmatory study found that the decoction pieces in three classical prescriptions of TCM emerging in the form of pharmacological groups and symptomatic treatment have clear evidence of pharmacological activity intensity; and pharmacodynamic effects in clinical practice against the signs and clinical manifestations of patients infected with COVID-19. For the clinical manifestations of COVID-19, such as body surface fever, cough, expectoration and wheezing of the respiratory system; gastrointestinal discomfort, poor stool, nausea, vomiting symptoms of the digestive system; less urine, blood urine of the urinary system, blood stasis, cardiopalmus, abdominal pain, myalgia and irritability caused by disease, the prescription components of TCM have an obvious symptomatic therapeutic effect in the form of pharmacological groups.(Figure 3)and (Table S2).

**Table S2:** Chinese herbal slices with corresponding pharmacological effects for symptoms and vital organs of COVID-19

Organs and tissues	Clinical manifestations or pathological changes	Proportion of patients with clinical manifestations	Symptomatic treatment	Pharmacological intensity of the components of TCM in Qingfei Paidu Decoction	The system frequency	Pharmacological intensity of the components of TCM in Huashi Baidu Decoction	The system frequency	Pharmacological intensity of the components of TCM in Hanshi Zufei Decoctio	The system frequency
Body surface	Fever	83-99%	Antipyretic	7	10	2	3	3	4
			Sweat	3		1		1	
	Cough	59-82%	Relieving a cough	9		2		2	
Respiratory system	Expectoration and blood-stained sputum		Eliminating phlegm	5	17	2	8	4	12
	Chest tightness/dyspnea and shortness of breath	31-40%	Relieving asthma	3		4		6	
			Promoting gastrointestinal digestive fluid secretion	0		0		4	
			Promoting gastric emptying	0		0		3	
	Gastrointestinal symptoms	39.60%	Repairing stomach damage	16		6		7	
Gastrointestinal system			Repairing intestinal damage	0	28	0	19	2	28
			Promoting bile secretion	3		1		2	
	inhibited defecation		Promoting gastrointestinal movement	7		10		8	
	Unformed stool		Anti-diarrhea	0		0		1	
	Nausea Vomiting	17.30% 5%	Stopping vomiting	2		2		1	
	Oliguria		Diuresis	8		5		2	
Urinary system	Urine is red		Anti-nephrotic changes (renal protection)	1	9	2	7	0	2
Hepatobiliary			Liver protection	10	13	7	8	4	6
system			Cholagogue	3		1		2	
Blood system			Anti-platelet aggregation	5	13	6	9	5	8
			Blood circulation	8		3		3	
			Cardioprotective effects	4		3		2	
The heart			Adrenergic action	2	10	3	10	4	10
	Palpitation		Antiarrhythmic effect	0		1		2	
			Cardiotonic effect	4		3		2	
	pain/myalgia Abdominal	11-35%	Analgesic effect	6		6		5	
Central system	Dysphoria		Sedation and anti-convulsion	4	10	1	7	0	7
			Antidepressant like effect	0		0		2	

Note: The Pharmacological intensity is the sum of the respective components of the three TCM prescriptions.

## Discussion

During the SARS outbreak in 2003, TCM carried out beneficial exploration. Pulmonary index, inflammatory factors, and inflammatory exudates improved significantly. In addition, it can also significantly reduce symptoms and promote the absorption of inflammation. It has also been actively explored in the treatment of COVID-19 and has achieved considerable results.

The treatment process of lung infection in “Jinkui Yaolue (Golden Chamber Synopsis) and “Shanghan Lun (Treatise on Febrile Diseases)” is actually consistent with the disease classification diagnosis process and treatment method in “Therapeutic Guidelines: Respiratory (Version 5)”. Gastrointestinal inflammation and cardiovascular dysfunction were also consistent. Diagnosis of the occurrence and development of diseases, identification of the etiology and status of a disease to give therapeutic measures, and the prescriptions of TCM and western medicine are consistent from the perspective of pharmacotherapy and pharmacology.

The treatment of TCM is based on traditional Chinese medicine, which is based on natural substances accumulated in clinical practice for a long time, including plants, minerals, and animals. Modern medical treatment is mainly based on drugs, and the basis of its usage is the accumulation of clinical treatment effects and pharmacological experiments. Modern Chinese medicine has carried out a large number of pharmacological experiments on the pharmacology of traditional Chinese medicine, and has achieved quite fruitful and positive results. A natural medicine that has undergone preliminary processing of TCM, has the characteristics of various components and pharmacological targets. The diversity and complexity of the prescriptions make it impossible to clarify a one-to-one pharmacodynamic relationship. In fact, if the multi-component, multi-chemical, multi-target and multi-pharmacological components of TCM are presented in the form of drug groups, pharmacological groups, and pharmacodynamic groups, it can present clear correspondence and therapeutic thinking, and we can see the comparability and relative unity of TCM and western medicine. Most COVID-19 patients have fever, cough, fatigue, anorexia, shortness of breath, myalgia and other nonspecific symptoms, such as sore throat, stuffy nose, headache, diarrhea, nausea and vomiting^54^.

Pharmacological activity of TCM only represents the existence of certain effects, but does not account for the strength of effects, which requires clear quantitative indicators. A comprehensive evaluation of the intensity of pharmacological action was performed based on the literature. Quantitative studies on the pharmacological effects of TCM can directly reflect the strength of the efficacy of TCM prescriptions. After the establishment of the dose-effect relationship model, the classical prescriptions of TCM showed a typical dose-effect relationship, and the corresponding relationship between the three classical prescriptions and the development of disease symptoms was clearly presented.

In the face of the surge of the omicron variant virus, it is necessary for us to further study the treatment of traditional Chinese medicine. From the severity analysis of the epidemic, the outbreak of multisystemic inflammatory cytokines is the most important step in the progression to crisis. Current treatments and experimental drugs approved by WHO Solidarity Trials are in fact mainly anti-inflammatory factor bursts.

Standard care includes corticosteroids and interleukin-6 receptor blockers, and casirivimab and imdevimab are add-on to current standard of care. Interleukin-6 receptor blockers can reduce mortality^55^ but they are expensive and currently difficult to access in many parts of the world. Etesevimab, a newly approved monoclonal antibody drug has treatment failure in Omicron^56^ . Long-term use of large doses of steroids such as dexamethasone can cause co-infection, especially superinfections^57^.

The three classic TCM prescriptions in this study produced clear and potent anti-inflammatory effects in the form of pharmacological groups supported by clear pharmacological evidence. The side effects of high-dose hormone therapy in the treatment of COVID-19 are decreased immunity, and secondary infections, including fungal infections, which are another focus and difficulty in the treatment of COVID-19.

Two years after the COVID-19 outbreak, several cases of severe acute respiratory syndrome developed. SARS-CoV-2, the variant of a new coronavirus, shows increased infectivity or immunity escape. Omicron (B.1.1.5229) is a variant of concern and was first identified in South Africa^58^ in November 2021. Omicron and Delta have been shown to have significantly improved advantages, extremely high transmission rates, and significant morbidity. The main treatment for the COVID-19 variant remain to kill the virus, isolate, treat symptomatically and improve the immune system. We can protect more lives if we use powerful and effective symptomatic treatments to maintain and stabilize organ function and use effective methods to combat the storm of inflammatory factors in the face of viral variants and turbulence. Standardized treatment of TCM, combined with the high-dose hormone therapy and antibody drug therapy of western medicine, is a great blessing for patients, and can make a great cost reduction and huge harvest for social management.

## The limitations of this study

Due to the limitation of our research level, the research on the corresponding relationship between TCM syndrome classification and Western medicine disease classification lacks further exploration; and simple description is not accurate enough.

In order to facilitate digital statistics and analysis, we measured the strength of pharmacology by grade intensity, which is an imperfect correspondence. And as a new concept, it has “immaturity”. In view of the limitations of the literature, some pharmacological effects that may be of great significance may have been omitted.

## Conclusion

Four crucial coronavirus outbreaks have been seen over the past 20 years, and scientific evidence and ecological reality suggest that coronavirus will also re-emerge in the future^59^. In the face of the re-emergence of coronavirus and the gap period of vaccines, drugs, and treatment methods for virus mutation, TCM can play a positive role in symptomatic treatment, alleviate symptoms and patients' suffering. Its strong and effective anti-inflammatory effects can protect more lives. Therefore, we hope TCM can bring some hope for the COVID-19 epidemic.
